# Cerebral Abscess

**Published:** 1875-03-15

**Authors:** D. A. K. Steele

**Affiliations:** Late Resident Physician, Cook County Hospital


### CEREBRAL ABSCESS—HISTORY—DEATH—AUTOPSY.

BY D. A. K. STEELE, M.D., LATE RESIDENT PHYSICIAN, COOK COUNTY HOSPITAL.

THE following case came under my observation while an *interne* in the County Hospital, and may present some features of interest to readers of the EXAMINER.

Michael Ganning, æt. about fifty-five; laborer; native of Ireland; was brought to the hospital in an unconscious condition on the afternoon of August 19th. Learned from friends that he had been drinking heavily for some weeks, lying around in a stupid condition, supposed to be superinduced by his continued debauch.

On admission, patient lies in a semi-comatose condition; breathing somewhat stertorous, unable to protrude his tongue; eyes sunken and congested; pupils contracted; face expressionless; lips and mouth dry, and coated with sordes. There is partial paralysis of right arm and leg, most marked in the arm. Physical examination of the chest reveals harsh respiration. A sharp, metallic click follows the first sound of the heart at the base. Patient is unable to swallow, showing paralysis of the pharyngeal muscles. Pulse 78, full and hard. Temp. 102½°; resp. 36.

Ordered bowels to be moved by an enema, and directed the nurse to endeavor to give him some liquid nourishment, milk, and beef-tea.

Aug. 20th, A.M.—Pulse 120; temp. 103°; resp. 42. Bowels moved. Unable to take any nourishment. Directed milk, per rectum.

P.M.—Pulse 168; temp. 104°; resp. 48.

Aug. 21, A.M.—Body assuming a cyanotic appearance. Pulse 168, very weak; temp. 104½°; resp. 60. Urine passed involuntarily.

P.M.—Symptoms increasing in gravity; died at 3 o'clock.

Post-mortem examination, eighteen hours after death.

Rigor mortis marked. Body cyanotic.

On removing calvarium, find the superficial veins of the brain distended with venous blood.

On removing the brain, with the exception of the superficial hyperæmia above noted, its general contour seems normal, except at the inferior portion of the middle lobe of the left hemisphere, just where it lies in apposition with the petrous portion of the temporal bone, and immediately in front of the tentorium cerebelli, where a portion of the brain substance about two inches in diameter was found quite soft. While examining the softened portion, a quantity of pus escaped from what seemed to be an abscess.

On section from above of the right hemisphere, it seems normal, with the exception of a small quantity of pus in the right lateral ventricle, which was afterwards found to have escaped from the abscess.

On section of the left hemisphere, found all that portion above the lateral ventricle normal in consistence and appearance.

Left lateral ventricle found filled with pus, and communicating with the abscess.

On section below the lateral ventricle, found the brain substance very much congested and softened, and in the inferior portion of the middle lobe, found an abscess about one and a half inches in diameter, communicating with the left lateral ventricle, from which the pus escaped externally, and into the ventricles, as previously noted. The cerebellum seemed healthy. A moderate amount of sero-sanguinous effusion was found at the base of the brain.

On opening the chest, found firm pleuritic adhesions of both lungs. In the right chest, extending from the second rib, and attached to the seventh, about two inches below the costo-sternal articulation, found an ossified plate about an inch wide and six inches long, curved at its inferior extremity, where it was attached to the seventh rib—it apparently had been developed in the costal pleura. Has no pathological significance. Lungs healthy. Pericardium thickened and adherent to the pleura. Heart weighed thirteen ounces. Mitral and aortic valves thickened. Left ventricle dilated slightly. Spleen small. Liver normal in size and appearance. Kidneys intensely congested; each weighed six ounces. Other organs not examined.

